# Perspectives in Wearable Systems in the Human–Robot Interaction (HRI) Field

**DOI:** 10.3390/s23198315

**Published:** 2023-10-08

**Authors:** Tao Liu, Xiangzhi Liu

**Affiliations:** The State Key Laboratory of Fluid Power and Mechatronic Systems, School of Mechanical Engineering, Zhejiang University, Hangzhou 310027, China; liuxiangzhi@zju.edu.cn

**Keywords:** highly integrated system, non-invasive measurement, wearable system, human–robot interaction (HRI)

## Abstract

Due to the advantages of ease of use, less motion disturbance, and low cost, wearable systems have been widely used in the human–machine interaction (HRI) field. However, HRI in complex clinical rehabilitation scenarios has further requirements for wearable sensor systems, which has aroused the interest of many researchers. However, the traditional wearable system has problems such as low integration, limited types of measurement data, and low accuracy, causing a gap with the actual needs of HRI. This paper will introduce the latest progress in the current wearable systems of HRI from four aspects. First of all, it introduces the breakthroughs of current research in system integration, which includes processing chips and flexible sensing modules to reduce the system’s volume and increase battery life. After that, this paper reviews the latest progress of wearable systems in electrochemical measurement, which can extract single or multiple biomarkers from biological fluids such as sweat. In addition, the clinical application of non-invasive wearable systems is introduced, which solves the pain and discomfort problems caused by traditional clinical invasive measurement equipment. Finally, progress in the combination of current wearable systems and the latest machine-learning methods is shown, where higher accuracy and indirect acquisition of data that cannot be directly measured is achieved. From the evidence presented, we believe that the development trend of wearable systems in HRI is heading towards high integration, multi-electrochemical measurement data, and clinical and intelligent development.

## 1. Introduction

Compared with traditional nursing care, the assistive robot (AR) [1,2] presents the advantages of professional rehabilitation methods, stable rehabilitation effects, and digital rehabilitation evaluation, which improves the accuracy and speed of the process of caregiving. Human–robot interaction (HRI) [3], a discipline that involves the interaction between humans and robots, is gradually being applied in clinical rehabilitation scenarios. Through the highly friendly HRI [4,5], the adaptation performance of AR to the patient’s rehabilitation process can be increased, and the poor effect or even damage caused by insufficient interaction can be reduced. Therefore, a sensing system [6] that can obtain the multimodal data of the human body quickly and with high precision is one of the critical links to improve the effect of HRI.

Due to its irreplaceable portability, research on wearable systems applied to the HRI field has become prevalent. Wang monitored the stride length, gait cycle, and other gait parameters of healthy people and patients in real-time using only two IMUs attached to two shanks and used the method of support vector machine (SVM) to realize the classification between patients and healthy subjects [7,8], which was also used to control the velocity of AR to suit patient gait. Falla et al. used a needle ECG measurement system to analyze the characteristics of the sternocleidomastoid muscle unit of nine women with chronic neck pain and nine healthy subjects and found that chronic neck pain affects the change in neural drive to muscles with force direction [9]. Winkert et al. used a wearable system to record the changes in oxygen and carbon dioxide of 12 participants during cycling under the different modes of breath-by-breath (BBB) and dynamic micro-mixing chamber (DMC) in order to compare the results with consecutive Douglas bag measurements. Finally, the reliability of the wearable system for measuring human exercise metabolism was verified [10]. Using three wearable sensors placed on the chest, thigh, and calf, Chen et al. [11] designed three rehabilitation modalities, including short-arc exercises, straight leg raises, and quadriceps-strengthening mini-squats for the assessment and management of knee osteoarthritis patient rehabilitation.

However, there are still some shortcomings in the wearable systems that have been used in the HRI field in the past. Firstly, the integration and flexibility of the measurement system were insufficient, resulting in the inability to fit the measurement surface well. Furthermore, due to the low integration, an additional power supply was often required (otherwise, the battery life could not guarantee the long-term measurement requirements), which increased the weight and complexity of the system and could not make good use of the portable characteristics [12]. Secondly, at present, many wearable systems still measure at the macro level (to be precise, limb movement signals such as speed and displacement or thoracic movement signals such as breathing) without going deep into molecular level analysis such as bodily fluid analysis, which obviously cannot meet the needs of in-depth HRI and comprehensive assessment in rehabilitation scenarios [13]. In addition, some physiological measurement methods in the clinical field, such as blood testing, ECG testing, etc., are primarily based on the invasive way to improve the signal-to-noise ratio, which significantly reduces the convenience of measurement and is not conducive to daily long-term monitoring.

**Figure 1:**
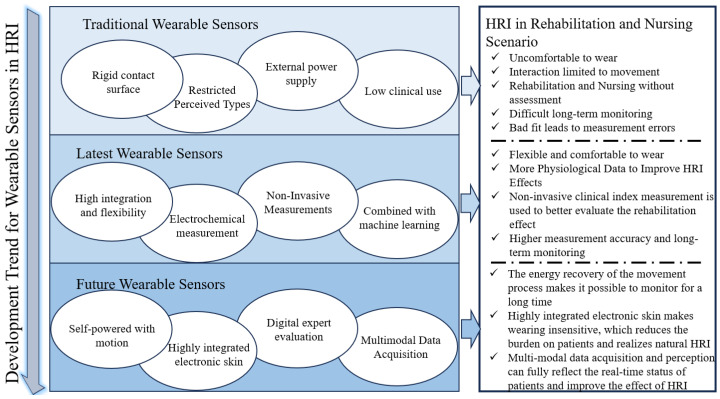
The development trends and perspectives of wearable systems in the HRI field.

How to solve these problems has become the future development direction of wearable systems used in the HRI field. As shown in Figure 1, many scholars have carried out a significant amount of exploratory research on these problems and solved these problems to a certain extent. This paper will review the existing research progress and achievements from four aspects and investigate the future development direction of the wearable systems used in the HRI field. Section 2 mainly concerns the progress of integration and flexibility of the wearable systems used in the HRI field. The current research is introduced, which combines the latest material production and high computing power chips to achieve a high fit, strong battery life, and compact structure. Section 3 mainly focuses on the latest progress of the wearable systems in electrochemical measurement. Through structural innovation and sensing unit design, the extraction and analysis of human bodily fluids such as sweat and tears are realized. Section 4 mainly focuses on non-invasive physiological measurement in clinical applications, which can further improve the adaptability of HRI to clinical scenarios. The studies that combine machine-learning methods to improve data accuracy and indirectly obtain previously unavailable data are described in Section 5. Section 6 summarizes the above work and investigates the future development direction of the wearable systems used in the HRI field.

## 2. Highly Integrated and Flexible Wearable Systems

The wearable systems used in the HRI field often require close contact with the measurement surface. Otherwise, the gap between the instrument and the measurement surface will often cause significant errors, especially for some weak signals (such as EMG). Since the measurement surface of the measured object, that is, the human body, is often irregular, there is a high requirement for the flexibility of the instrument’s measurement area, which is in direct contact with the human body. However, simultaneously, the measured signal needs to be amplified and preprocessed by subsequent chips and circuits before it can be used for evaluation, and the current mainstream chips and circuits are still rigid. In addition, wearable systems are always powered by an external power supply, such as lithium batteries, which inevitably increase the system’s weight and reduces portability. Therefore, using the latest progress in materials and innovative structural design to integrate power supply, processing chip, and flexible sensor into one system, where adjustable fit, ease of wear, and the assurance of battery life can be achieved, has become a significant development focus. At present, many studies have achieved breakthroughs in this regard.

**Figure 2:**
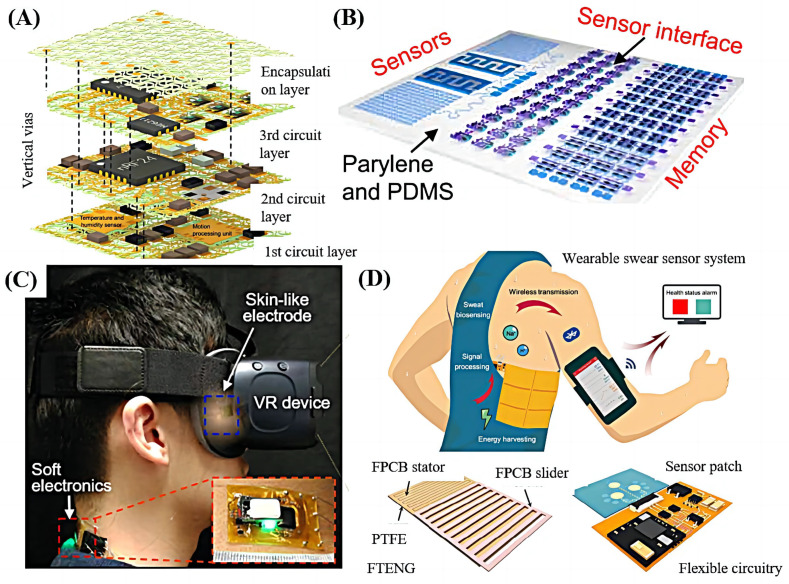
The highly integrated and flexible wearable system. (**A**) Schematic illustration of the device system in an exploded view. The system contains 42 ICs, with lateral dimensions of 11.2 mm by 10.1 mm and an areal coverage ratio of 110%. Subcircuits are aligned and electrically connected through vertical vias, highlighted by dashed lines [14]. Reproduced with permission from Song, H., Science Advance; published by AAAS, 2022. (**B**) Spatial layout of the whole system and the architecture of sensors, sensor interface circuits, and an integrated flash memory array (24 bit by 16 bit) [17]. Reproduced with permission from Xiang, L., Science Advance; published by AAAS, 2022. (**C**) A portable, wearable system for vergence detection via skin-like electrodes and soft circuit with a VR system [18]. Reproduced with permission from Mishra, S., Science Advance; published by AAAS, 2020. (**D**) Schematic illustrating the system that integrates human motion energy harvesting, signal processing, microfluidic-based sweat biosensing, and Bluetooth-based wireless data transmission to a mobile user interface for real-time health status tracking [20]. Reproduced with permission from Song, Y., Science Advance; published by AAAS, 2020.

As shown in Figure 2A, Song et al. [14] designed a highly integrated and flexible electronic system based on stacked multilayer network materials (SMNM), which realized high-precision sensing of human body temperature, humidity, and nine-degree-of-freedom motion. Compared with previously reported inorganic electronic systems with similar levels of biaxial elastic stretchability and functional complexity [15,16], the designed system offers a much smaller lateral dimension, higher area coverage ratio, and better elastic stretchability.

Epidermal electronic systems can simultaneously provide the acquisition, processing, and storage of biological signals, an urgent requirement for future healthcare, diagnostic, and clinical applications. Many studies have used the latest integration techniques and material properties to achieve applications in these areas. As shown in Figure 2B, based on carbon nanotube (CNT) thin films, Xiang et al. [17] built an epidermal system including flexible sensors (humidity, temperature, and electrocardiogram sensors), sensor interface circuits (high-performance differential amplifiers), and integrated elastic flash memory arrays (24 bit vs. 16 bit), which were used for physiological information recording. The weak biological signals were amplified by high-performance differential amplifiers (voltage gain 27 dB, common-mode rejection ratio > 43 dB, gain-bandwidth product > 22 kHz), and the processed information was stored in the memory array. The findings revealed the excellent application potential of epidermal electronic systems in personalized diagnosis and physiological monitoring. As shown in Figure 2C, Mishra et al. [18] developed a fully wearable wireless soft electronic system through the combination of a skin conformal sensor and virtual reality system that could completely fit the contour surface of the nose and surrounding eyes and collect electrooculography (EOG) with high precision. A portable, high-sensitivity eye movement tracking system was implemented and validated on 14 subjects.

In addition, due to the pursuit of small size and light weight, the integration technology of wearable systems will inevitably face the challenge of battery life. Many studies have adopted different solutions for the battery life problem in system integration. Tehrani et al. [19] designed a miniaturized, fully integrated, wirelessly operated microneedle technology to continuously monitor interstitial fluid (ISF) biomarkers in freely behaving human participants. Through the battery optimization method, the battery life of the wearable system could reach up to 30 days. As shown in Figure 2D, Song et al. [20] innovatively solved the endurance problem by effectively extracting energy from body motion through a flexible printed circuit board (FPCB)–based freestanding triboelectric nanogenerator (FTENG). Compared with traditional triboelectric nanogenerators (TENGs), it showed remarkable mechanical and electrical stability, even in large-scale commercial manufacturing processes after intensive mechanical deformation and repeated washing cycles.

Although current research has made technological breakthroughs in the integration, flexibility, and long battery life of wearable systems, there is still a certain distance from the ideal HRI in AR systems. In terms of integration, although the integration of acquisition, processing, and storage modules has been achieved through structural optimization and material innovation, there needs to be more verification of the working stability of highly integrated wearable systems in harsh environments and long-term use. Moreover, the battery life breakthrough is more appropriate for small systems, and there is still a gap in improving the battery life of the whole body measurement system (such as electronic skin). There is still a particular gap between this and clinical scenarios where the patient’s wearable system achieves the goal of being separated from external power sources and has the ability of monitoring data for long periods of time.

## 3. Wearable Systems for Electrochemical Measurement

In the past, the wearable systems used in the HRI field were more frequently at the macro level and did not dive deep into the micro level, such as regarding molecules. The parameters at the macro level can only evaluate the external movement of the person, and it is difficult to perceive and assess the changes in the patient’s physiological indicators, which limits the rehabilitation and nursing level of AR [21]. While analysis of bodily fluids is ideal for a comprehensive evaluation of the human body, among common biological fluids such as sweat, tears, saliva, and ISF, sweat is especially popular because it is readily available from the skin surface and contains abundant markers such as metabolites (e.g., glucose, lactate), proteins, and hormones, which all offer excellent potential for biometric-oriented wearable systems.

**Figure 3:**
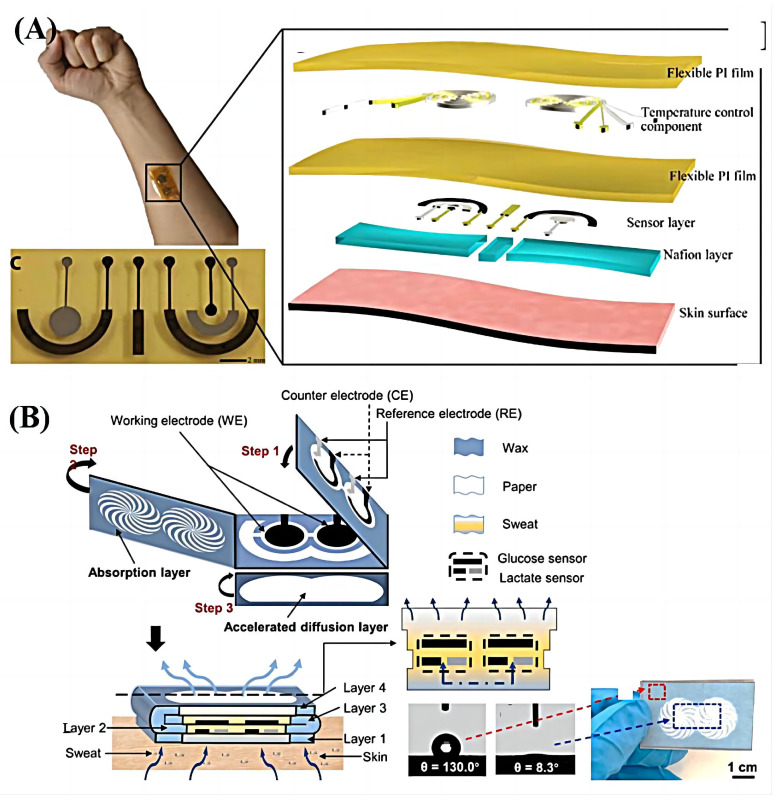
Wearable system for electrochemical measurement. (**A**) Photo of the real application of the proposed device and detailed structure of the integrated epidermal biomicrofluidic device [22]. Reproduced with permission from Pu, Z., Science Advance; published by AAAS, 2017. (**B**) Structural anatomy of HIS paper and photographs of the foldable HIS paper [24]. Reproduced with permission from Li, M., Biosensors and Bioelectronics; published by Elsevier (Amsterdam, The Netherlands), 2021.

At present, many researchers are committed to extracting and analyzing specific components in biological fluids (usually sweat) and then evaluating the physiological state of the human body. As shown in Figure 3A, Pu et al. [22] designed a flexible electronics-based epidermal bio-microfluidic technology for clinical continuous glucose monitoring. The influence of passive perspiration is eliminated through a differential structure, improving blood glucose measurement accuracy. Moreover, the system adopts a complete printing process that is easy to manufacture and low in cost. Some researchers have combined the measurement results of single components in sweat with macro-level assessment. Hong et al. [23] designed a multifunctional wearable health management system consisting of a sweat-based glucose sensing strip and a wearable smart band. The multilayered structure of the sweat-analysis strip enables efficient sweat collection and visualization of the collected sweat. In addition to analyzing sweat glucose levels, the integrated system can continuously monitor vital signs (i.e., heart rate, blood oxygen saturation level, and physical activity), realizing the function of estimating blood glucose caused by physical activity.

However, more researchers still want to realize the multi-parameter measurement of biological fluid through only one wearable system. As shown in Figure 3B, Li et al. [24] designed a low-cost, freestanding, and disposable highly integrated sensing paper (HIS paper). Through the innovative design of the multi-layer structure of the HIS paper, the efficient collection and component separation of human bodily fluid (sweat) is realized. HIS paper has been appllied to analyze human bodily fluid glucose and lactate levels, with sensitivities of 2.4 μAmMH${}^{-1}$ and 0.49 μAmMH${}^{-1}$, respectively. Gao et al. [25] designed a mechanically flexible and fully integrated (embedded processing chip) sensor array for human sweat analysis. By merging commercially available integrated circuit technology, the system integrated different sensing modules to realize the study of metabolites (such as glucose and lactic acid) and electrolytes (such as sodium and potassium ions) in sweat. He et al. [26] also designed a flexible sweat analysis patch based on silk-derived carbon textiles for the simultaneous detection of glucose, lactate, ascorbic acid, uric acid, $Na^{+}$, and $K^{+}$ in sweat.

Most of the electrochemical composition analysis of the human body is based on wet electrodes, which often inevitably leads to system inconvenience, instability, and human infection, which could be more conducive to long-term signal monitoring. Therefore, some researchers have pioneered analyzing human bodily fluid components based on dry electrodes [27]. Bio-impedance (BioZ) measurement has been increasingly used in human bodily fluid composition analysis, and Shu et al. [28] verified that the measured BioZ settles faster and reaches the level of wet electrodes under the high input impedance of dry electrodes. The possibilities for the future development of dry electrode-based BioZ sensors are illustrated.

In the electrochemical measurement of wearable systems, three key points still need to be paid attention to based on the current research. One is the long-term monitoring of the level of electrochemical changes in the human body, which is of great significance in terms of evaluating the human body. The second is the control of system cost and the low threshold of mass production in terms of the devices that will replace the existing human body electrochemical measuring instruments in the future. The third is using the perceived physiological data to establish a patient’s rehabilitation evaluation model and then adjust the AR rehabilitation plan in real time to improve clinical care.

## 4. Clinical Applications of Non-Invasive Physiological Measurements

Physiological measurement has always played a vital role in clinical medicine. Accurate physiological measurement results can help doctors better understand the patient’s status in order to design an accurate and personalized treatment plan. However, most physiological measurement methods currently used in clinical practice are non-wearable and invasive, such as invasive blood drawing, which cannot realize portable monitoring and requires high-cost laboratory analysis. In this way, when the AR system is used in rehabilitation scenarios, patients will inevitably experience pain, discomfort, and the risk of pathogen infection, significantly reducing the rehabilitation effect. With the continuous development of wearable system technology, many researchers are committed to replacing traditional clinical invasive instruments with non-invasive wearable systems in appropriate scenarios in order to alleviate patient discomfort.

**Figure 4:**
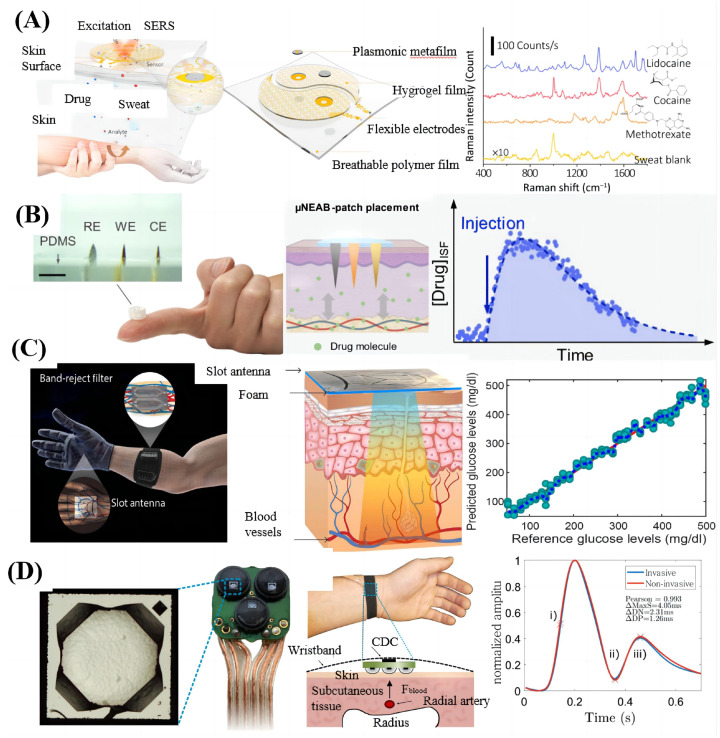
Clinical applications of non-invasive physiological measurements. (**A**) Schematic drawing showing the working principle and design of the device (**left**) and SERS spectra of the human sweat samples containing different drug (**right**) [29]. Reproduced with permission from Wang, Y., Science Advance; published by AAAS, 2021. (**B**) Photos of an assembled μNEAB−patch, consisting of microneedle electrodes embedded in an elastomeric substrate; WE, CE, and RE, respectively, denote working, counter, and reference electrodes (**left**) and in vivo continuous readouts are used to infer the drug’s amount (**right**) [30]. Reproduced with permission from Lin, S., Science Advance; published by AAAS, 2022. (**C**) The proposed sensors operate at the upper ultrahigh frequency and microwave bands, ensuring enough electromagnetism wave penetration depth (**left**) and the non-invasively estimated glucose versus the reference glucose (**right**) [31]. Reproduced with permission from Hanna, J., Science Advance; published by AAAS, 2020. (**D**) Photograph of the square-shaped element with a side length of 1.2 mm (**left**) and Pearson correlation coefficient between the invasive and non-invasive measurements (**right**) [32]. Reproduced with permission from Kaisti, M., NPJ digital medicine; published by Springer Nature (Berlin/Heidelberg, Germany), 2019.

The correct dosage and timing of medication are critical to achieving precise and personalized drug therapy. Especially for some drugs with a narrow therapeutic window (such as antibiotics), if the level of the drug in the patient’s body cannot be captured in time, the drug will not be able to act at the optimal time and may even harm the patient. Some researchers have achieved the monitoring of drug levels through non-invasive physiological measurement methods that provide additional input for HRI control in the clinic. As shown in Figure 4A, Wang et al. [29] designed a wearable plasmonic sensor with universal molecular recognition capabilities. Combined with a flexible electronic sweat extraction system, the metabolites in vivo, based on its unique surface-enhanced Raman scattering (SERS) spectroscopy, can be non-invasively extracted and processed. The wearable system successfully monitors drug changes in the body and obtains individual drug metabolism profiles. As shown in Figure 4B, Lin et al. [30] prepared a microneedle-based electrochemical aptamer biosensing patch (μNEAB−patch) to minimally detect ISF and correlate the pharmacokinetics of circulating drugs with continuous and real-time measurement. Different types of μNEAB−patch were prepared for different medications, including antibiotics (tobramycin and vancomycin), and their functions were verified in animals.

Diabetes is a notable problem in current clinical medicine. Over the past few decades, invasive continuous blood glucose measurement methods have made significant progress, but pain and discomfort limit the possibility of daily blood glucose measurement. Fortunately, many studies have achieved technological breakthroughs in non-invasive blood glucose measurement. As shown in Figure 4C, Hanna et al. [31] proposed a first-of-its-kind, high-sensitivity, non-invasive wearable multi-sensor system for continuous blood glucose monitoring. The non-invasive acquisition of human blood glucose has been realized through two electromagnetic sensors with frequency bands between 500 MHz and 3 GHz and a circuit structure that simulates the human blood vessel system. The high correlation between the blood glucose level measured by the system and the reference blood glucose level has been verified (>0.9) without delay. Continuous cardiovascular health monitoring is of great significance to the clinical treatment of cardiovascular diseases. As shown in Figure 4D, Kaisti et al. [32] designed a flexible wearable wristband based on an array of microelectromechanical sensor (MEMS) elements. Through the wristband, an MEMS pressure sensor element capable of measuring absolute pressure in the range of 0–120 kPa is placed on the wrist, forming a three-layer structure of carpal bone-artery-sensor to realize the pulse wave measurement. Comparing the system results with the results of invasive measurement equipment (industry gold standard), the pulse waveform is almost the same (r = −0.9–0.99). Sustained sleep spike (CSWS) syndrome is one of the most common epileptic encephalopathies in children. Carvalho et al. [33] used a wearable device that could record two bipolar EEG channels for 24 h to simplify the quantification of peak index, replacing the traditional method of repeated 10–20 long-term dynamic EEG analysis. It was verified on 38 patients, and the effects of different treatment options were confirmed.

These researchers have achieved breakthroughs in the clinical application of wearable systems, but there still needs to be further clinical research to investigate certain aspects of these systems. The first aspect is sample size. The current research is more frequently laboratory sample research, requiring more samples to support the results. The second is the interpretation of results. The current clinical field needs not only a convenient wearable system to obtain data but also a framework or model to interpret the data and then evaluate and even predict the physiological state of the patients so that clinicians can diagnose and adjust the treatment plans of AR.

## 5. Wearable Measurement System Combined with Machine Learning

Physiological measurements in HRI can quantify various indicators of patients and adjust AR rehabilitation care plans. However, many results or parameters related to the human body still need to be measured through commonly used wearable systems (such as the incidence of diseases or the recognition of exercise patterns). With the development of machine-learning technology, many studies can directly construct undetermined mathematical models between human body data measured by wearable systems and target results, realizing the acquisition of initially difficult results.

**Figure 5:**
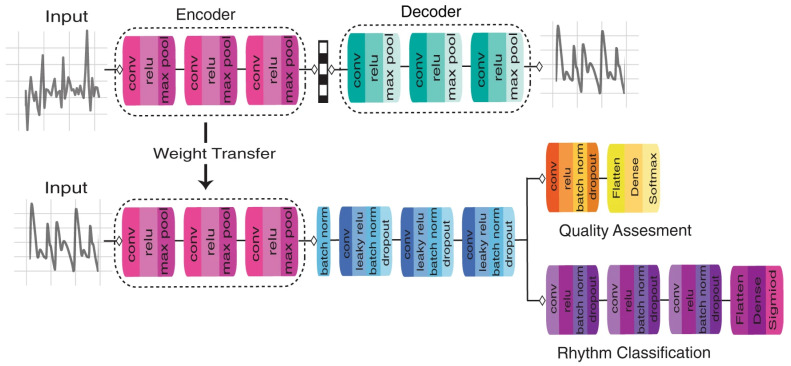
Physiological measurement system combined with machine learning. The proposed model architecture for DeepBeat. The top represents the pretraining process on the unlabeled simulated data, and the bottom represents the multitask fine-tuning process on the labeled data [35]. Reproduced with permission from Torres-Soto., NPJ digital medicine; published by Springer Nature, 2020.

Combining machine-learning methods with wearable systems to predict patient clinical outcomes has become a research focus. Bini et al. [34] used three wrist-based activity trackers to collect data from 22 total joint arthroplasty patients. Thirty-five features were collected from the patient from 4 weeks before surgery to 6 weeks after surgery. The system predicts patient measurements over six weeks after total joint arthroplasty through machine-learning methods. Monitoring physiological measurements by wearable photoplethysmography devices has great potential but is challenging to implement due to noise effects. As shown in Figure 5, Torres-Soto et al. [35] developed a multi-task deep-learning method called DeepBeat to detect atrial fibrillation (AF) events in real time based on wrist-based photo plethysmography (PPG) sensing. The algorithm utilizes a multi-task CNN structure, transfer learning, and auxiliary tasks to perform signal quality estimation for AF event detection from spatially segmented physiological PPG signals, addressing the common unique noise artifacts in AF detection. Wicaksono et al. [36] proposed a low-power, wearable, convenient gastric data acquisition (g-DAQ) system to identify gastric content images in the epigastric region, where sectoral electrical impedance tomography (s-EIT) and a K-means sectoral clustering algorithm were used. The system was applied to measure the emptying process of 15 subjects to evaluate the retention level of gastric contents.

Based on the above work, the wearable system has successfully realized the indirect acquisition and prediction of physiological parameters through machine learning, but it still faces the following challenges. The first is the universality of the algorithm. How to avoid over-fitting the results so that the method can adapt to different situations is still the focus of future research. The second is the accuracy of the results. Due to the diversity of the combination of eigenvalues and selection of the undetermined mathematical model, the accuracy of physiological parameter measurements is also variable. Finding the optimal parameter configuration and improving the output accuracy of the algorithm are still important research directions that are needed in order for the system to be applied to actual physiological measurements.

## 6. Discussion

Existing perspectives and some review papers describe the development of wearable sensors in human–computer interaction and other aspects, to a certain extent. For example, Harito et al. [37] reviewed the recent technological progress of intelligent polymer systems, which have further integrated and strengthened the power supply module of wearable devices and have significant application potential for long-term clinical monitoring. Similarly, Liu et al. [38] also proposed a perspective on wearable sensor bodily fluid measurement, demonstrating the clinical value of wearable devices in terms of hardware materials, long-term wear, and data reliability. Cui et al. [39] comprehensively discussed the application of microstructured flexible pressure sensors in wearable human–computer interaction and ex vivo and in vivo health monitoring.

However, the above research only shows the application of wearable sensors in a particular field, and it does not help readers fully understand the current research status of combining wearable sensors with other areas. Moreover, most of the relevant research was published three or more years ago, while many of the research results selected in this article are from the past two years, which can better reflect the latest research progress of wearable sensors in the field of human–computer interaction. Furthermore, this article reviews wearable sensors in highly integrated and flexible hardware, electrochemical measurement, non-contact and clinical applications, and machine-learning-driven measurement methods. There are vital issues that need to be solved regarding actual human–computer interaction.

This paper also has certain shortcomings. First, this article is only a brief review of wearable sensors in human–computer interaction. It only includes recent essential works to put forward a future inference rather than draw accurate conclusions. Secondly, although this article covers the application status of four major categories of wearable sensors, there is still a lack of discussion on other requirements worthy of concern, such as privacy protection and cost control of data collected by wearable sensors, which are still crucial for practical application scenarios.

## 7. Conclusions and Perspective

In recent years, with the rapid development of MEMS manufacturing technology and new materials, wearable systems have achieved technological breakthroughs in non-invasive, high-integration, electrochemical measurement, and machine-learning methods, making wearable systems more suitable for clinical treatment assistance and daily health monitoring.

The future development of wearable systems used in the HRI field can be summarized regarding the following aspects. The first is to develop a highly integrated and flexible wearable system with high working stability and long battery life. With the continuous development of new battery technology and flexible material manufacturing technology, a set of electronic skin that can fully fit the human body and monitor human physiological parameters in different environments for a long time is bound to become the mainstream of future research(of course, there are still many technical obstacles to implementing electronic skin, such as longer system battery life). The combination of electronic skin and AR in rehabilitation care scenarios makes long-term monitoring of patient data and intelligent rehabilitation possible. The second is the more vital significance of clinical applications. Given the limitations of traditional clinical devices and the insufficiency of the current sensing system, based on realizing non-invasiveness, the present patient classification diagnosis (whether the patient is sick or the current drug level) is transitioned to a more in-depth assessment (the degree of the patient’s disease or real-time medication regimen adjustment). Quantitative assessment rather than classified diagnosis makes the HRI-based AR rehabilitation program transition from type adjustment to intensity change in clinical scenarios. Finally, it is more closely integrated with machine-learning methods. The combination of machine-learning methods can improve the system’s accuracy and anti-noise ability and build an undetermined mathematical model between the measurement data and the target data to realize the indirect acquisition of data that could not be directly measured previously.
